# Interactions of genetic variants reveal inverse modulation patterns of dopamine system on brain gray matter volume and resting-state functional connectivity in healthy young adults

**DOI:** 10.1007/s00429-015-1134-4

**Published:** 2015-10-25

**Authors:** Jiayuan Xu, Wen Qin, Bing Liu, Tianzi Jiang, Chunshui Yu

**Affiliations:** 1 Department of Radiology and Tianjin Key Laboratory of Functional Imaging, Tianjin Medical University General Hospital, No. 154, Anshan Road, Heping District, Tianjin, 300052 China; 2 Brainnetome Center, Institute of Automation, Chinese Academy of Sciences, Beijing, 100190 China; 3 National Laboratory of Pattern Recognition, Institute of Automation, Chinese Academy of Sciences, Beijing, 100190 China

**Keywords:** Catechol-*O*-methyltransferase, Dopamine D2 receptor, Functional magnetic resonance imaging, Gray matter volume, Resting-state functional connectivity

## Abstract

Different genotypic combinations of *COMT* and *DRD2* can generate multiple subgroups with different levels of dopamine signaling. Its modulations on brain properties can be investigated by analyzing the combined gene effects of *COMT* and *DRD2*. However, the inherent association between modulation patterns of the dopamine system on structural and functional properties of the brain remains unknown. In 294 healthy young adults, we investigated both additive and non-additive interactions of *COMT* and *DRD2* on gray matter volume (GMV) and resting-state functional connectivity (rsFC) using a voxel-based analysis. We found a significant non-additive *COMT* × *DRD2* interaction in the right dorsal anterior cingulate cortex (dACC), exhibiting an inverted U-shape modulation by dopamine signaling. We also found a significant non-additive *COMT* × *DRD2* interaction in the rsFC between the right dACC and precuneus, displaying a U-shape modulation by dopamine signaling. Moreover, this rsFC was negatively correlated with the GMV of the right dACC. Although the additive interaction did not pass corrections for multiple comparisons, we also found a trend towards an inverse modulation pattern and a negative correlation between the GMV and rsFC of the right inferior frontal gyrus. No genotypic differences were detected in any assessments of the cognition, mood and personality. These findings suggest that healthy young adults without optimal dopamine signaling may maintain their normal behavioral performance via a functional compensatory mechanism in response to structural deficit due to genetic variation.

**Electronic supplementary material:**

The online version of this article (doi:10.1007/s00429-015-1134-4) contains supplementary material, which is available to authorized users.

## Introduction

As an important neurotransmitter, dopamine is involved in the modulation of the cognitive and emotional processing (Girault and Greengard 2004; Seamans and Yang 2004). The dopamine system affects human behaviors via modulating brain structural and functional properties in a nonlinear manner (Williams and Goldman-Rakic 1995, 1998; Seamans and Yang 2004; Bertolino et al. 2009). The dopamine signaling in the brain depends on both the availability of dopamine and the efficiency of dopamine receptors. The availability of dopamine can be modulated by the catechol-*O*-methyltransferase (COMT), which catalyzes the degradation of synaptic dopamine, especially in the prefrontal cortex (PFC) (Männistö and Kaakkola 1999; Seamans and Yang 2004). *COMT* gene contains a functional polymorphism (rs4680, G > A), resulting in a decrease in enzymatic activity in *A*-*allele* carriers (Männistö and Kaakkola 1999), through which *COMT* polymorphism can modulate structure and function of the brain that affect individuals’ behavioral performance. The efficiency of dopamine receptors is also modulated by genetic variation. A functional polymorphism (rs1076560, G > T) of the dopamine D2 receptor (DRD2) gene can modulate *DRD2* efficiency by modifying the ratios of its two isoforms (Zhang et al. 2007).

According to the genotypes of *COMT rs4680* and *DRD2 rs1076560*, one can approximately estimate the dopamine signaling in the brain. The *COMT* *rs4680*-*GG* subjects have greater *COMT* activity and lower dopamine signaling than the *A*-*allele* carriers (Matsumoto et al. 2003). For the *DRD2 rs1076560*, *GG* genotype has greater expression of D2S (inhibiting dopamine release) and lower dopamine signaling than *T*-*allele* carriers (Zhang et al. 2007). Consequently, individuals with the *rs4680*-*GG*/*rs1076560*-*GG* status may have the lowest dopamine signaling; in contrast, individuals with the *rs4680*-*A*-*allele/rs1076560*-*TT* status may have the highest dopamine signaling. In this frame, we can explore the non-linear modulation of the dopamine system on structure and function of the brain by observing combined effects between *COMT* and *DRD2* genotypes.

The effects of the common genetic variants of *COMT* and *DRD2* on behavioral performance and brain structural and functional properties have been originally described in populations with European ancestry (Bruder et al. 2005; Egan et al. 2001; Mattay et al. 2003; Meyer-Lindenberg et al. 2006; Zhang et al. 2007). This knowledge has been subsequently translated to Asia populations (Li et al. 2009; Liu et al. 2010; Ohnishi et al. 2006; Zhang et al. 2007; Zheng et al. 2012). However, most of these studies focus on the respective effects of *COMT* or *DRD2* (Taylor et al. 2007; Cerasa et al. 2008, 2009, 2010; Honea et al. 2009; Li et al. 2009; Ehrlich et al. 2010; Liu et al. 2010). Only a few studies have focused on *COMT* × *DRD2* interactions on behavioral performance (Reuter et al. 2005, 2007; Xu et al. 2007). A recent study has explored *COMT* × *DRD2* interactions on functional connectivity density (FCD) in healthy subjects and found completely inversed nonlinear modulation patterns of the dopamine signaling on the FCDs of the different functional systems (a “control system” and a “processing system”), suggesting a functional system-dependent modulation of dopamine signaling (Tian et al. 2013). However, it is unclear whether there are *COMT* × *DRD2* interactions on structural properties of the brain in healthy subjects. If so, we further want to know whether the dopamine signaling exhibits similar or contrary modulation patterns on structural and functional properties of the brain. Similar modulation patterns mean that a subgroup with structural deficit would also have functional deficit, predicting worse behavioral performance in this subgroup. In contrast, contrary modulation patterns mean that a subgroup with structural deficit would exhibit functional enhancement, predicting a nearly normal behavioral performance.

To answer these questions, we performed a series of exploratory analyses in the 294 healthy young Chinese Han subjects. The additive gene–gene interactions were analyzed by linear regression, and the main effect of each SNP and the non-additive gene–gene interactions were analyzed by a two-way analysis of variance (ANOVA). First, we assessed main effects, additive and non-additive interactions of *COMT* and *DRD2* on behavioral performance of the cognition, mood and personality domains. Second, we used gray matter volume (GMV) as a measure of brain structural properties and investigated the modulation of the dopamine signaling on GMV by analyzing *COMT* × *DRD2* interactions. Third, we used resting-state functional connectivity (rsFC) as a measure of brain functional properties. We investigated nonlinear modulation of the dopamine signaling on these functional connections by analyzing *COMT* × *DRD2* interactions. Only rsFCs of brain regions with significant interaction effects on GMV were included to maintain the regional consistency between the structural and functional analyses. Finally, a direct correlation between GMV and rsFC in any of brain regions with significant interactions on both measures was assessed in the total population of subjects.

## Materials and methods

### Subjects

A total of 323 healthy young Chinese Han adults (mean age 22.7 ± 2.5 years; 157 males and 166 females) participated in this study. Participants were carefully screened to ensure that they had no history of psychiatric or neurological illness, drug or alcohol abuse, and that they had no contraindications to MRI examination. All subjects were strongly right-handed according to the Chinese edition of the Edinburgh Handedness Inventory (Oldfield 1971). The study was approved by the Medical Research Ethics Committee of Tianjin Medical University, and all participants provided written informed consent. Memory Quotient was tested using the Chinese Revised Wechsler Memory Scale (WMS-RC) (Gong 1989) and executive function was assessed using the Stroop Color-Word Task (MacLeod 1991) and Wisconsin Card Sorting Test (WCST) (Heaton 1999). Individual working memory capacity was evaluated by the *n*-back task (Owen et al. 2005). Depression and anxiety levels were evaluated with the beck depression inventory (BDI) (Beck et al. 1996) and the Self-Rating Anxiety Scale (SAS), respectively (Zung 1971). The Tridimensional Personality Questionnaire (TPQ) was used to assess personality dimensions (Cloninger et al. 1993).

### Genotyping

We extracted genomic DNA from 3000 µL of whole blood using the EZgeneTM Blood gDNAMiniprep Kit (BiomigaInc, San Diego, CA, USA). Subjects’ genotypes for *COMT rs4680* and *DRD2 rs1076560* were determined using the PCR and ligation detection reaction (LDR) method (Thomas et al. 2004; Yi et al. 2009) with technical support from the Shanghai Biowing Applied Biotechnology Company. PCR primer sequences were as follows: forward of *COMT*: 5′ GGGCCTACTGTGGCTACTCA 3′; reverse of *COMT*: 5′ CCCTTTTTCCAGGTCTGACA 3′; forward of *DRD2*: 5′ AGCATCTCCATCTCCAGCTC 3′; and reverse of *DRD2*: 5′ GAAAAAGGACAGGGGCAATC 3′. PCR was performed with a 20 μL reaction volume containing 1 μL genomic DNA, 0.4 μL primer mixture, 2 μL dNTPs, 0.6 μL Mg^2+^, 2 μL buffer, 4 μL Q-Solution, and 0.3 μL Taq DNA polymerase. The amplification protocol incorporated an initial denaturation and enzyme activation phase at 95 °C for 15 min, followed by 35 cycles of denaturation at 94 °C for 30 s, annealing for 1 min and 30 s at 59 °C for *COMT* rs4680 and 56 °C for *DRD2 rs1076560*, extension at 72 °C for 1 min, and then a final extension at 72 °C for 7 min. PCR products were verified in 3 % agarose gels that had been stained with ethidium bromide to regulate the amount of DNA added to the LDR.

For each SNP, three probes were designed for the LDR reactions: one common probe (*rs4680*: P-GCCAGCGAAATCCACCATCCGCTGGTTTTTTTTTTTTTTTTTTTT-FAM; *rs1076560*: P-GAAAGGGAGGGGCCAGTGAGATGGGTTTTTTTTTTTTTTTTTT-FAM) and two discriminating probes for the two alleles of each SNP (*rs4680_A*: TTTTTTTTTTTTTTTTTTTTCAGGCATGCACACCTTGTCCTTCAT; *rs4680_G*: TTTTTTTTTTTTTTTTTTTTTTCAGGCATGCACACCTTGTCCTTCAC; *rs1076560_T*: TTTTTTTTTTTTTTTTTTGTGTTTGCAGGAGTCTTCAGAGGGA; *rs1076560_G*: TTTTTTTTTTTTTTTTTTTTGTGTTTGCAGGAGTCTTCAGAGGGC). These reactions were conducted in a 10 μL mixture containing 1 μL buffer, 1 μL probe mix, 0.05 μL Taq DNA ligase, 1 μL PCR product, and 6.95 μL deionized water. The reaction program consisted of an initial heating at 95 °C for 2 min, followed by 35 cycles of 30 s at 94 °C and 2 min at 50 °C. Reactions were stopped by chilling the tubes in an ethanol-dry ice bath and adding 0.5 mL of 0.5 mM EDTA. Aliquots of the reaction products (1 μL) were mixed with 1 μL of loading buffer (83 % formamide, 8.3 mM EDTA and 0.17 % blue dextran) and 1 μL ABI GS-500 Rox-Fluorescent molecular weight marker and then denatured at 95 °C for 2 min.

The samples were then chilled rapidly on ice prior to being loaded on a 5 Murea-5 % polyacrylamide gel and electrophoresed on an ABI 3100 DNA sequencer at 3000 V. Finally, the fluorescent ligation products were analyzed and quantified using the ABI Gene Mapper software. Twenty-eight of the 323 subjects were excluded due to genotyping failure. The remaining 295 healthy young adults with both *COMT* and *DRD2* genotypes were ultimately included for the future analysis.

### Image acquisition

MR imaging was acquired using a Signa HDx 3.0 Tesla MR scanner (General Electric, Milwaukee, WI, USA). Sagittal 3D T1-weighted images were acquired by a brain volume (BRAVO) sequence with the following parameters: repetition time (TR)/echo time (TE) = 8.1/3.1 ms; inversion time = 450 ms; field of view (FOV) = 256 mm × 256 mm; matrix = 256 × 256; flip angle (FA) = 13°, slice thickness = 1 mm; no gap; 176 sagittal slices. Resting-state functional MRI (fMRI) data were collected using Single-Shot Echo-Planar Imaging (SS-EPI) (TR/TE = 2000/30 ms; FOV = 240 mm × 240 mm; matrix = 64 × 64; FA = 90°, slice thickness = 4 mm; no gap; 40 interleaved transverse slices; 180 volumes). During the fMRI scans, all subjects were instructed to keep still with their eyes closed, to think of nothing in particular, to stay as motionless as possible, and to not fall asleep. After the fMRI scan, fMRI images and subjects’ conditions were checked to confirm whether they satisfied the requirement. If not, the fMRI data were abandoned and scanned again.

### Data preprocessing

All structural images were visually checked by two experts separately. One of the remaining 295 subjects was excluded due to poor image quality; thus, a total of 294 subjects were included in the voxel-based morphometry (VBM) analysis. GMV maps were constructed for each subject using the VBM8 implemented in Statistical Parametric Mapping software package (SPM8, http://www.fil.ion.ucl.ac.uk/spm). The structural MR images were segmented into gray matter (GM), white matter (WM) and cerebrospinal fluid (CSF) using a new segmentation model in the VBM8. The new segmentation model is an extension of the “unified segmentation” algorithm, which includes additional tissue probability maps to better model CSF and other non-brain voxels, resulting in a more accurate segmentation (Ashburner and Friston 2005). Following segmentation, GM population templates were generated from the entire image dataset using the diffeomorphic anatomical registration through the exponentiated Lie algebra (DARTEL) technique (Ashburner 2007). After an initial affine registration of the GM template to the tissue probability map in the Montreal Neurological Institute (MNI) space, non-linear warping of GM images was performed to the GM template in the MNI space with a resolution of 1.5 × 1.5 × 1.5 mm^3^ (as recommended for the DARTEL procedure). The GMV of each voxel was obtained by multiplying the GM concentration map by the non-linear determinants derived from spatial normalization procedure. Finally, the GMV images were smoothed with a full width at half maximum (FWHM) kernel of 8 × 8 × 8 mm^3^ to compensate for residual anatomical differences between subjects. Thus, the regional GMV represents normalized GMV after removing the confounding effect of variance in individual brain sizes. After spatial pre-processing, the normalized, modulated, and smoothed GMV maps were used for statistical analysis.

The fMRI data were preprocessed using the Data Processing Assistant for Resting-State fMRI (DPARSFA) (Chao-Gan and Yu-Feng 2010). The first ten volumes for each subject were discarded to allow the signal to reach equilibrium and the participants to adapt to the scanning noise. The remaining 170 volumes were then corrected for the acquisition time delay between slices. Fourteen subjects were excluded from further analysis because their fMRI data had a maximum displacement in one or more of the orthogonal directions (*x*, *y*, *z*) of >2 mm or a maximum rotation (*x*, *y*, *z*) >2.0°. Thus, a total of 280 subjects were included in the rsFC analysis. We also calculated framewise displacement (FD), which indexes volume-to-volume changes in head position. These changes were obtained from the derivatives of the rigid-body realignment estimates that are used to realign blood oxygen level-dependent (BOLD) data during fMRI preprocessing (Power et al. 2012, 2013). There was no main effect of each SNP and interaction effect on FD (*P* > 0.05). All data were then spatially normalized to the standard EPI template and resampled to a voxel size of 3 × 3 × 3 mm^3^. The normalized data were smoothed by a Gaussian kernel of 6 × 6 × 6 mm^3^. After normalization, several nuisance covariates (six estimated motion parameters and average BOLD signals of the whole brain and ventricular and white matter regions) were removed from the data using a regression analysis. Finally, the datasets were band-pass filtered with frequency from 0.01 to 0.1 Hz.

### Statistical analysis

Statistical analyses for demographic and psychological data were performed using the Statistical Package for the Social Sciences version 18.0 (SPSS, Chicago, IL, USA) for Windows. The additive gene–gene interactions were analyzed by linear regression, and the main effect of each SNP and non-additive gene–gene interactions were analyzed by a two-way ANOVA. These analyses were used to evaluate the main effect of each SNP and their interactions for the demographic and psychological data.

Voxel-based GMV comparisons were performed using a two-way (*COMT* and *DRD2*) ANOVA with the age, gender and years of education as nuisance variables. Multiple comparisons were corrected using the Gaussian random field (GRF) method with a voxel level threshold of *P* < 0.005 and a cluster level threshold of *P* < 0.005. Brain regions that showed a significant *COMT* × *DRD2* interaction effects on GMV were extracted and defined as seed regions for the following rsFC analysis. The detailed methods for additive gene–gene interactions on GMV are provided in Supplementary materials.

For each subject, the correlation coefficient between the mean time series of the seed region and that of each voxel in the whole brain (except for the seed region) was computed and transformed into a *z* value to improve normality. Subsequently, individuals’ *z* values were entered into a random effects one-sample *t* test to identify brain regions exhibiting significant positive correlations with the seed region. Significant rsFC maps were corrected for multiple comparisons with the Family Wise Error (FWE, *P* < 0.05) method. And then, a two-way (*COMT* and *DRD2*) ANOVA was applied within a mask of brain areas with significant positive rsFCs with the seed region. Multiple comparisons were corrected using the GRF method with the same thresholds as being used in the GMV comparisons.

We used quadratic regression to test the significance of the fitting curve that modeling the modulation of the presumed dopamine signaling on the GMV and rsFC of brain regions with a significant interaction effect. Different sorting schemes were tested. By sorting according to *COMT*, the presumed dopamine signaling from low to high is the *rs4680*-*GG/rs1076560*-*GG*, *rs4680*-*GG/rs1076560*-*GT*, *rs4680*-*GG/rs1076560*-*TT*, *rs4680*-*A*-*allele/rs1076560*-*GG*, *rs4680*-*A*-*allele/rs1076560*-*GT*, and *rs4680*-*A*-*allele/rs1076560*-*TT*. By sorting according to *DRD2*, the presumed dopamine signaling from low to high is the *rs1076560*-*GG/rs4680*-*GG*, *rs1076560*-*GG/rs4680*-*A*-*allele*, *rs1076560*-*GT/rs4680*-*GG*, *rs1076560*-*GT/rs4680*-*A*-*allele*, *rs1076560*-*TT/rs4680*-*GG*, and *rs1076560*-*TT/rs4680*-*A*-*allele.*

## Results

### Demographic and genetic characteristics

The demographic and genetic characteristics are shown in Table 1. The VBM analysis included 294 subjects and the rsFC analysis included 280 subjects. The distributions of *COMT rs4680* (139 *GG*, 125 *GA*, and 30 *AA*) and *DRD2 rs1076560* (109 *GG*, 136 *GT*, 49 *TT*) were both in Hardy–Weinberg equilibrium (*P* > 0.05). The *GA* and *AA* subjects were merged into one group of *A*-*allele* carriers due to the relatively low frequency of *AA* homozygotes (4–5 times lower than *GG* homozygotes); this method has been used previously to address skewed genotypic distributions (Taylor et al. 2007). Thus, these subjects were divided into six genotypic subgroups: *rs4680*-*GG/rs1076560*-*GG*, *rs4680*-*GG/rs1076560*-*GT*, *rs4680*-*GG/rs1076560*-*TT*, *rs4680*-*A*-*allele/rs1076560*-*GG*, *rs4680*-*A*-*allele/rs1076560*-*GT*, and *rs4680*-*A*-*allele/rs1076560*-*TT.* No genotypic distribution differences were found between males and females. We did not find any significant main or interaction effects for any demographic variables (*P* > 0.05). Neither significant main effects of any SNP nor significant additive or non-additive interactions between the two SNPs (*P* > 0.05) were found for any of the cognitive (memory and execution) and psychological variables (depression, anxiety and personality).

**Table 1:** Demographic data for VBM (*n* = 294) and rsFC (*n* = 280) analyses

Genotypic groups	*n*	Age (years)	Years of education	Gender (male/female)
VBM analysis	294			
*rs4680*-*GG/rs1076560*-*GG*	47	23.3 (2.3)	16.1 (2.0)	22/25
*rs4680*-*GG/rs1076560*-*GT*	66	22.6 (2.4)	15.7 (2.2)	33/33
*rs4680*-*GG/rs1076560*-*TT*	26	22.7 (2.5)	15.7 (1.8)	9/17
*rs4680*-*A*-*allele/rs1076560*-*GG*	62	22.0 (2.3)	15.2 (2.0)	33/29
*rs4680*-*A*-*allele/rs1076560*-*GT*	70	23.0 (2.5)	15.5 (2.4)	35/35
*rs4680*-*A*-*allele/rs1076560*-*TT*	23	22.7 (3.1)	15.8 (2.2)	10/13
Statistics		*F* = 2.02	*F* = 1.19	*χ* ^2^ = 2.97
*P*		0.07	0.31	0.71
rsFC analysis	280			
*rs4680*-*GG/rs1076560*-*GG*	45	23.3 (2.3)	16.1 (2.0)	20:25
*rs4680*-*GG/rs1076560*-*GT*	64	22.6 (2.4)	15.6 (2.2)	31:33
*rs4680*-*GG/rs1076560*-*TT*	26	22.7 (2.5)	15.7 (1.8)	9:17
*rs4680*-*A*-*allele/rs1076560*-*GG*	60	22.0 (2.3)	15.2 (2.0)	32:28
*rs4680*-*A*-*allele/rs1076560*-*GT*	64	22.9 (2.3)	15.5 (2.3)	31:33
*rs4680*-*A*-*allele/rs1076560*-*TT*	21	23.0 (3.0)	16.0 (2.1)	9:12
Statistic		*F* = 1.86	*F* = 1.29	*χ* ^2^ = 2.93
*P*		0.10	0.27	0.71

The data are shown as the means (SD)

*VBM* voxel-based morphometry, *rsFC* resting-state functional connectivity

### The non-additive interactions on GMV

Although we focused on the significant interaction effect between the two SNPs, there was also a significant main effect of *COMT* on the GMV (GRF correction at voxel level *P* < 0.005 and cluster level *P* < 0.005). However, there was no significant main effect of *DRD2* on GMV under the same statistical threshold. Detailed results are provided in Supplementary results and Fig. S1.

**Fig. 1:**
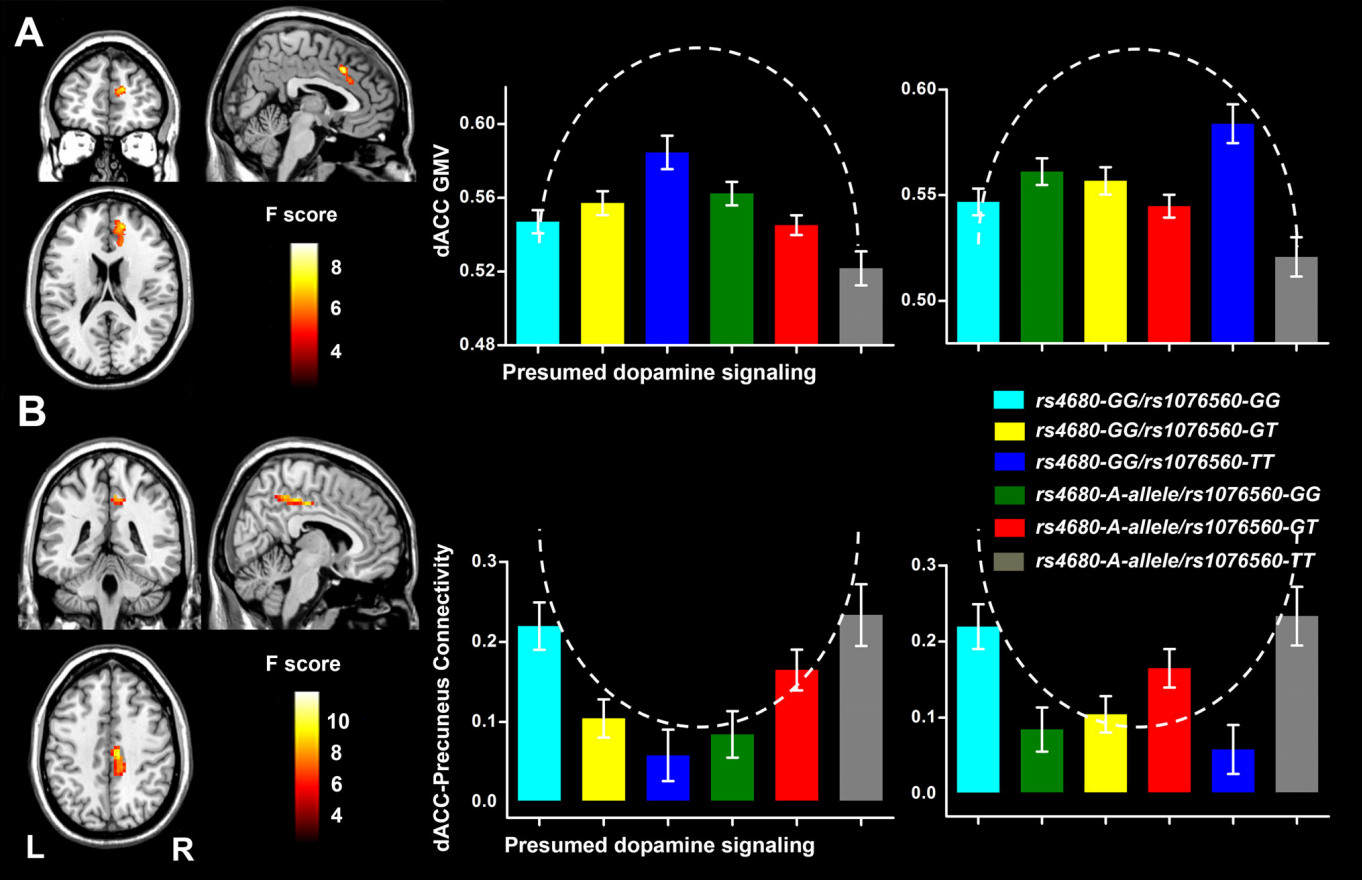
Brain regions with significant non-additive *COMT* × *DRD2* interactions on GMV (**a**) and rsFC (**b**). The *left* *column* shows the location of brain region with a significant non-additive interaction on GMV (**a**) or rsFC (**b**). The *mediate* and *right* *column* shows the modulation effect of the presumed dopamine signaling on GMV and rsFC by sorting genotypes according to *COMT* and *DRD2*, respectively. The *horizontal* *axis* of the *bar* *plot* represents six genotypic subgroups with presumed dopamine signaling from low to high. The *dashed* *line* represents the hypothesized dopamine modulation pattern (inverted “U” shape) on GMV of the right dACC and (“U” shape) its rsFC with precuneus. *COMT* catechol-*O*-methyltransferase, *dACC* dorsal anterior cingulate cortex, *DRD2* D2 receptor gene, *GMV* gray matter volume, *L* left, *R* right, *rsFC* resting-state functional connectivity

The GMV analysis revealed a significant non-additive *COMT* × *DRD2* interaction in the right dorsal anterior cingulate cortex (dACC) (Brodmann area 32; peak MNI coordinate: *x* = 4.5, *y* = 19.5, *z* = 39; 1060 voxels; peak *F* = 14.23) with a GRF correction for multiple comparisons (voxel level *P* < 0.005 and cluster level *P* < 0.005) (Fig. 1a). The GMV of the right dACC with a significant *COMT* × *DRD2* interaction was extracted from each subject. For each genotypic subgroup, the means and standard errors of GMV of the right dACC are shown in Fig. 1a. The distribution of the GMV of this cluster in these genotypic subgroups (which reflected the presumed dopamine signaling) was displayed as an inverted U-shape (Fig. 1a). By sorting according to *COMT*, the fitting curve tested by quadratic regression was significant (*r* ^2^ = 0.071, *P* = 0.00003) (Fig. S2A). By sorting according to *DRD2*, the fitting curve was still significant (*r* ^2^ = 0.036, *P* = 0.004) (Fig. S3A).

### The non-additive interactions on rsFC

**Fig. 2:**
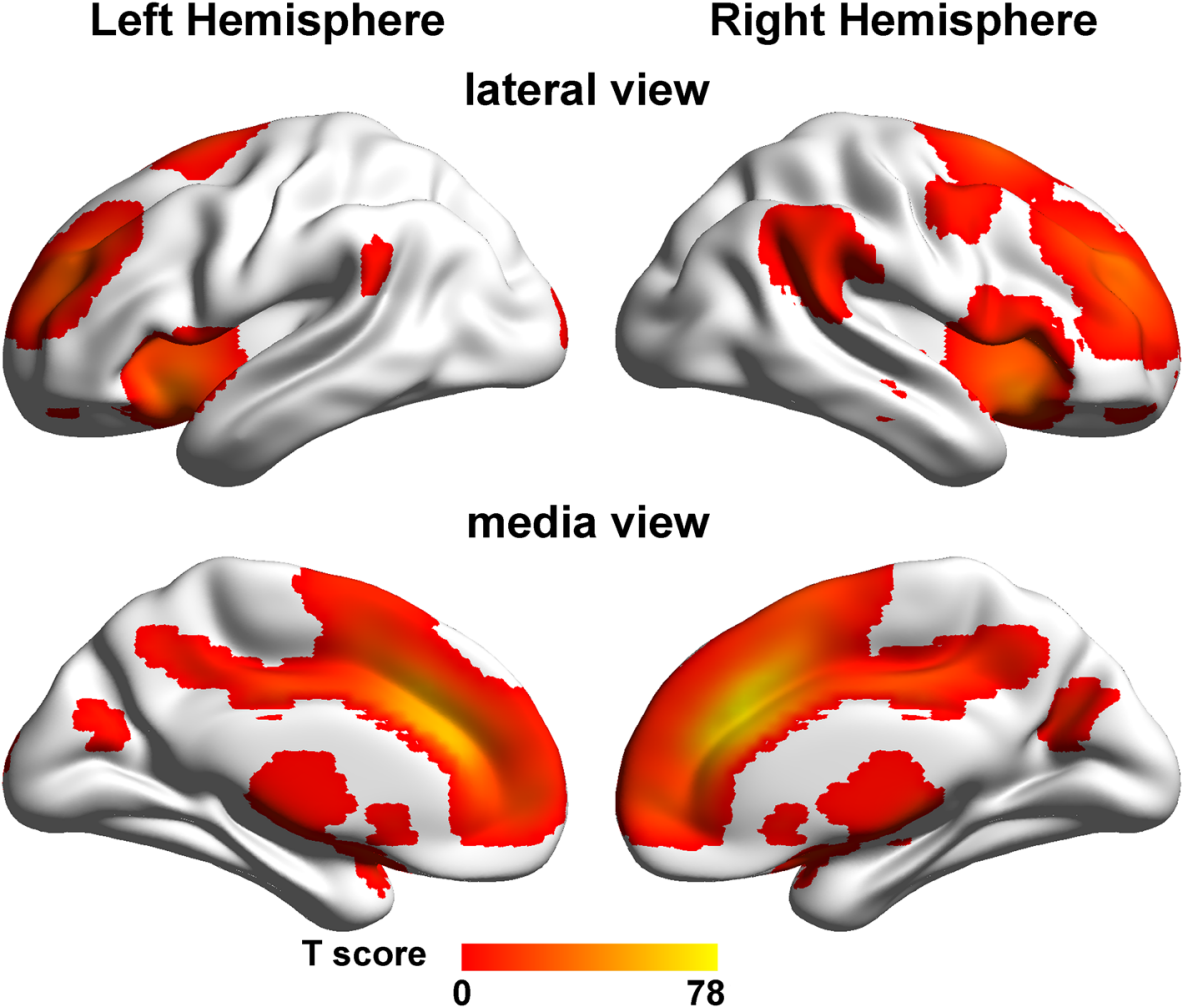
The rsFC maps of the right dACC. One-sample *t* test (FWE, *P* < 0.05) reveals that the right dACC is positively correlated with of brain regions of the salience network. *FWE* family wise error, *SN* salience network, *L* left, *dACC* dorsal anterior cingulate cortex, *R* right, *rsFC* resting-state functional connectivity

For the rsFC analysis, we defined the seed region as the cluster of the right dACC that showed a significant *COMT* × *DRD2* interaction on GMV. A one-sample *t* test (FWE, *P* < 0.05) in the total population revealed that the right dACC was positively correlated with brain regions of the salience network (SN) including the ACC, MCC, precuneus, lateral prefrontal cortex and frontoinsular cortex (FIC) (Fig. 2). These findings suggest that the right dACC is an important node of the SN.

Although we did not find any significant main effect of *COMT* or *DRD2* polymorphism on the rsFC of the right dACC (GRF correction at voxel level *P* < 0.005 and cluster level *P* < 0.005), we found a significant non-additive *COMT* × *DRD2* interaction on the rsFC between the right dACC and the right precuneus (Brodmann area 23; peak MNI coordinate: *x* = 9, *y* = −33, *z* = 45; 72 voxels; peak *F* = 11.51) (Fig. 1b). The rsFC between the right dACC and the right precuneus with a significant *COMT* × *DRD2* interaction was extracted from each subject. For each genotypic subgroup, the means and standard errors of the rsFCs of these subgroups are shown in Fig. 1b. The distribution of the rsFCs of these genotypic subgroups (which reflected the presumed dopamine signaling) was fitted into a U-shaped model (Fig. 1b). By sorting according to *COMT*, the fitting curve was significant (*r* ^2^ = 0.075, *P* = 0.00002) (Fig. S2B). By sorting according to *DRD2*, the fitting curve was still significant (*r* ^2^ = 0.048, *P* = 0.001) (Fig. S3B).

### Correlation between GMV and rsFC of the right dACC

**Fig. 3:**
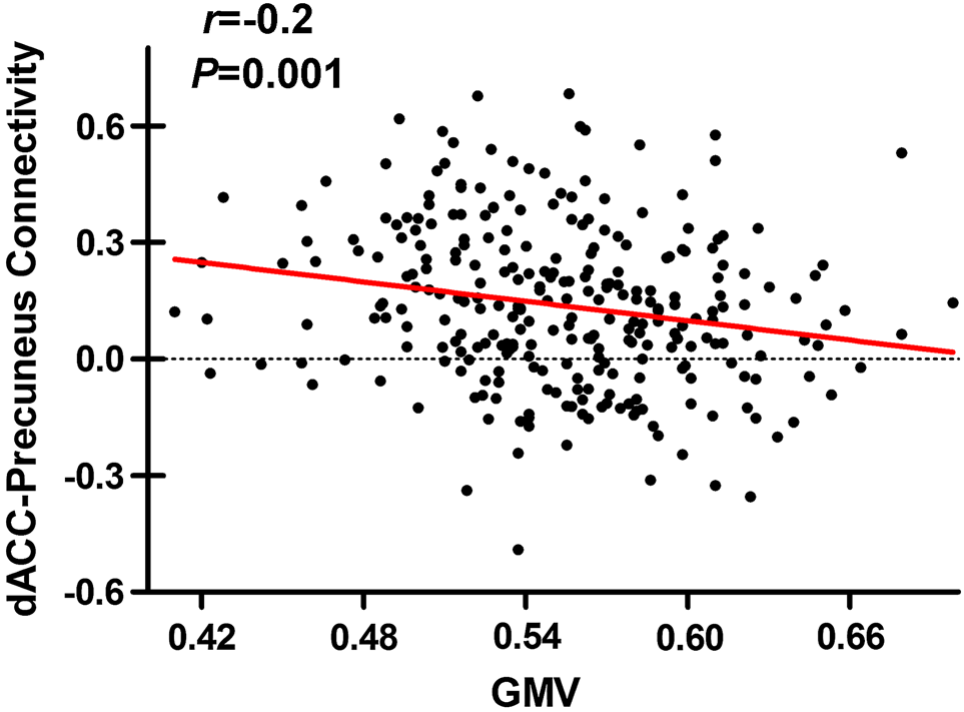
Correlation between GMV of the right dACC and rsFC between the right dACC and precuneus. *GMV* gray matter volume, *dACC* dorsal anterior cingulate cortex, *rsFC* resting-state functional connectivity

Investigation of correlation between GMV and rsFC of the right dACC may help to elucidate the structure–function relationship in brain regions that were modulated by dopamine-related genetic variation. Thus, we tested correlation between GMV of the right dACC and rsFC between the right dACC and the right precuneus. We found a significant negative correlation (*r* = −0.2, *P* = 0.001) between structural and functional measures of the right dACC (Fig. 3).

### The additive interactions on GMV

Although we did not find any significant additive interaction under the same statistical threshold (GRF correction at voxel level *P* < 0.005 and cluster level *P* < 0.005), we found a trend towards significant additive effects (*P* < 0.005, uncorrected) of the two SNPs on the GMV in the right inferior frontal gyrus (IFG) (BA 44, peak MNI coordinate: *x* = 34.5, *y* = 7.5, *z* = 19.5; 1191 voxels; peak *T* = −3.683). Post hoc testing revealed a linear modulation pattern: the GMV of right IFG decreased with increasing dopamine signaling (Fig. S4A).

### The additive interactions on rsFC

We then extracted the right IFG as seed region to perform rsFC analyses. A one-sample *t* test (FWE, *P* < 0.05) in the total population revealed that the right IFG was positively correlated with brain regions of the bilateral temporal and frontal lobes (Fig. S5). Voxel-based comparisons of rsFCs were then performed using a one-way ANOVA (*P* < 0.005, uncorrected) to identify brain regions with group differences. We found a significant group differences in the rsFC between the right IFG and the right superior temporal gyrus (STG) (Brodmann area 22; peak MNI coordinate: *x* = 66, *y* = −27, *z* = 15; 36 voxels; peak *F* = 5.100). Post hoc testing revealed that the rsFC between the right IFG and STG increased with increasing dopamine signaling (Fig. S4B).

### Correlation between GMV and rsFC of the right IFG

We tested correlation between GMV of the right IFG and rsFC between the right IFG and STG. We found a significant negative correlation (*r* = −0.12, *P* = 0.042) between structural and functional measures of the right IFG (Fig. S6).

## Discussion

In this study, we explored modulation patterns of the dopamine system on structural (GMV) and functional (rsFC) properties of the brain in healthy young adults by investigating additive and non-additive interactions between *COMT* and *DRD2*. We found an inverse modulation of the dopamine system on the GMV and rsFC of the brain. The GMV showed an inverted U-shaped modulation, whereas the rsFC showed a U-shaped modulation. Moreover, structural and functional properties in these brain regions showed a negative correlation in this population.

In the present study, we found a typical inverted U-shaped modulation of the dopamine signaling on GMV of the right dACC. The ACC has rich dopamine innervations and *DRD2* distribution (Williams and Goldman-Rakic 1998), and the gray matter density of the ACC has been found to be positively correlated with the expression of D2-like receptors (Woodward et al. 2009). Although the exact mechanisms of the inverted U-shaped modulation of the dopamine signaling on GMV remain unclear, a potential mechanism is dopamine signaling-dependent neurotropic and neurotoxic effects (Honea et al. 2009). The dependence of neuronal survival and growth on dopamine signaling has been described as an inverted U-shaped curve. In this model, an optimal extracellular dopamine signaling may induce the generation of brain derived neurotropic factor (BDNF) (Küppers and Beyer 2001) and facilitate neuronal growth. Conversely, either too low or too high extracellular dopamine signaling may impair neuronal integrity and survival (Santiago et al. 2000). For example, the BDNF expression is reduced in the frontal cortex of the dopamine transporter knockout mice with the higher extracellular dopamine signaling (Fumagalli et al. 2003). The dopamine D1 receptor mutant mice with the lower extracellular dopamine signaling shows deficient in dopamine-mediated behavioral response (Xu et al. 1994).

We also found a non-linear modulation of the dopamine signaling on rsFC between the right dACC and precuneus in healthy young adults. According to the rsFC pattern of the right dACC (Fig. 2), both the right dACC and precuneus were nodes of the salience network (SN). The SN is responsible to identify the most relevant among internal and extrapersonal stimuli and to initiate appropriate control signals to modulate higher order cognitive processes (Seeley et al. 2007; Menon and Uddin 2010). The dopamine system has been shown to play an important role in the function of the SN. In healthy subjects, dopamine has emerged as the primary neurochemical mediator in relation to various traits and behaviors mediated by the SN including prediction error signals, novelty-seeking, craving and nociception (Suhara et al. 2001; Contreras et al. 2007; Coffeen et al. 2008; Naqvi and Bechara 2010). Moreover, the dopamine system plays an important role in the modulation of structural and functional properties of the SN in healthy subjects. For example, the gray matter density in brain regions of the SN is positively correlated with the expression of D2-like receptors (Woodward et al. 2009), the functional connectivity density of the SN regions is nonlinearly modulated by the dopamine signaling (Tian et al. 2013), and the dopamine neurotransmission in the SN regions increases significantly during the performance of executive processes (Ko et al. 2009). The structural and functional deficits of the SN have also been found in disorders that are characterized by a dopaminergic abnormality. In schizophrenia patients, *COMT rs4680* *GG* homozygous patients (with lower dopamine signaling) have a significant GMV reduction in the SN regions compared to *A*-*allele* carriers (with higher dopamine signaling) (Ohnishi et al. 2006). In individuals with schizophrenia, abnormal dopaminergic transmission has been observed in the SN (Suhara et al. 2002; Takahashi et al. 2006), which may be associated with structural and functional deficits in the SN. For example, schizophrenia patients have exhibited reduced activation in the SN regions when performing working memory (Henseler et al. 2009) and error processing tasks (Polli et al. 2008). Methamphetamine releases dopamine from vesicular storage pools into the cytoplasm; excess dopamine may cause damage to dopaminergic fibers by its neurotoxic effect (Larsen et al. 2002). As expected, the methamphetamine abusers have shown the GMV reduction in the SN regions (Thompson et al. 2004).

The most important finding of this study is an inverse modulation of the dopamine system on structural and functional properties of the right dACC in healthy young adults, i.e., the GMV showed an inverted U-shaped modulation, whereas the rsFC showed a U-shaped modulation. As we aforementioned, the inverted U-shaped modulation of the dopamine signaling on GMV is well consistent with the inverted U-shaped model of the dependence of neuronal survival and growth on dopamine signaling (Küppers and Beyer 2001). We proposed a compensatory mechanism to explain for the inverse modulation of dopamine signaling on structural and functional properties of the brain in healthy young adults. Individuals with *rs4680*-*GG/rs1076560*-*TT* or *rs4680*-*A*-*allele/rs1076560*-*GG* genotype have optimal dopamine levels, which corresponds to normal GMV and rsFC of the right dACC. In these subjects, the normally developed structural and functional properties correspond to their normal behavioral performance. In contrast, individuals with *rs4680*-*GG/rs1076560*-*GG* or *rs4680*-*A*-*allele/rs1076560*-*TT* genotype have a dopamine signaling that is either too low or too high, which may impair neuronal integrity and survival (Santiago et al. 2000). This can explain for the decreased GMV in the right dACC in these subjects. However, the enhanced rsFC of the right dACC and normal behavioral performance in these subjects suggest a functional compensatory mechanism. That is, healthy young adults with abnormal dopamine signaling may maintain their normal behavioral performance via enhancing rsFC of the dACC in response to structural deficit of this region due to genetic variation. This inference is supported by our finding of a negative correlation between GMV and rsFC of the dACC in this population. Moreover, this hypothesis is also supported by our analysis of the additive effects that showed a trend towards an inverse modulation pattern and a negative correlation between the GMV and rsFC of the right IFG. The phenomenon of functional compensation for structural deficit due to genetic variation in healthy young subjects has also been observed in the *KIBRA* polymorphism that is related to Alzheimer’s disease. In healthy young subjects, *KIBRA C*-*allele* carriers have a smaller GMV and an increased functional connectivity in the cognitive-related brain regions than *TT* individuals (Wang et al. 2013).

It should be noted that the *COMT* is specifically related to the prefrontal cortex dopamine modulation (Akil et al. 2003), whereas the *DRD2* is much more important in the striatal dopamine modulation (Sambataro et al. 2013). The expression of *COMT* and *DRD2* in the ACC is not fully understood and the reciprocal relationship between brain structure and function is rather complex. Future studies should be done to validate our interpretation. Furthermore, since the allele frequencies of both SNPs vary greatly across ethnic populations (DeMille et al. 2002; Mukherjee et al. 2010; Palmatier et al. 1999), our findings derived from Chinese Han population should be validated in other ethnic populations.

## Conclusion

We investigated modulation patterns of the dopamine signaling on structural (GMV) and functional (rsFC) properties of the brain in healthy young adults and found inverse modulation patterns. The inverted U-shaped modulation of the dopamine signaling on structural property may be explained by the inverted U-shaped model of the dependence of neuronal survival and growth on dopamine signaling. However, the U-shaped modulation of the functional property may indicate a functional compensatory mechanism through which healthy young adults with structural deficits could maintain their normal behavioral performance. However, these findings should be confirmed in independent Chinese Han samples and in other ethnic populations, especially samples of European origin.

## Electronic supplementary material

Below is the link to the electronic supplementary material.

Supplementary material 1 (DOC 14935 kb)

Supplementary material 2 (DOC 36 kb)

Supplementary material 3 (DOC 34 kb)
