# Supplementary material for: Interactions of genetic variants reveal inverse modulation patterns of dopamine system on brain gray matter volume and resting-state functional connectivity in healthy young adults
Source: Brain Struct Funct. 2015 Oct 25;221(8):3891–901. doi: 10.1007/s00429-015-1134-4 (PMC5065899; doi:10.1007/s00429-015-1134-4)
Supplement: Supplementary file 1 — Supplementary material 1 (DOC 14935 kb) [file 429_2015_1134_MOESM1_ESM.doc]

**Supplementary Figures**

#
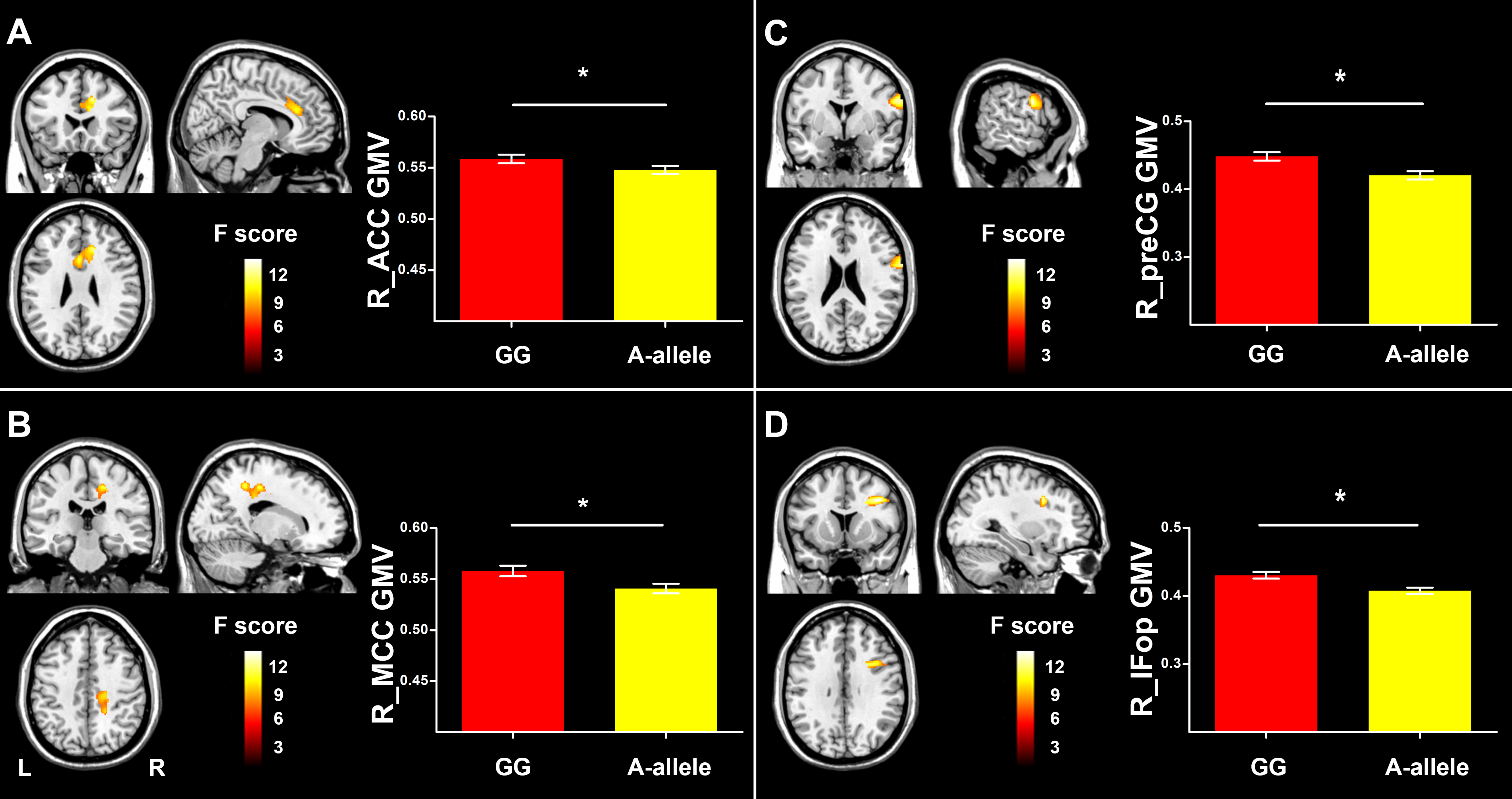


# Figure S1. The main effects of *COMT* on GMV. *COMT* A-allele carriers exhibit significantly (voxel level P < 0.005 and cluster level P < 0.005) smaller GMV in the right ACC, MCC, precentral gyrus and IFop than GG homozygotes. ACC, anterior cingulate cortex; COMT, catechol-O-methyltransferase; GMV, gray matter volume; IFop, inferior frontal operculum; L, left; MCC, middle cingulate cortex; R, right.


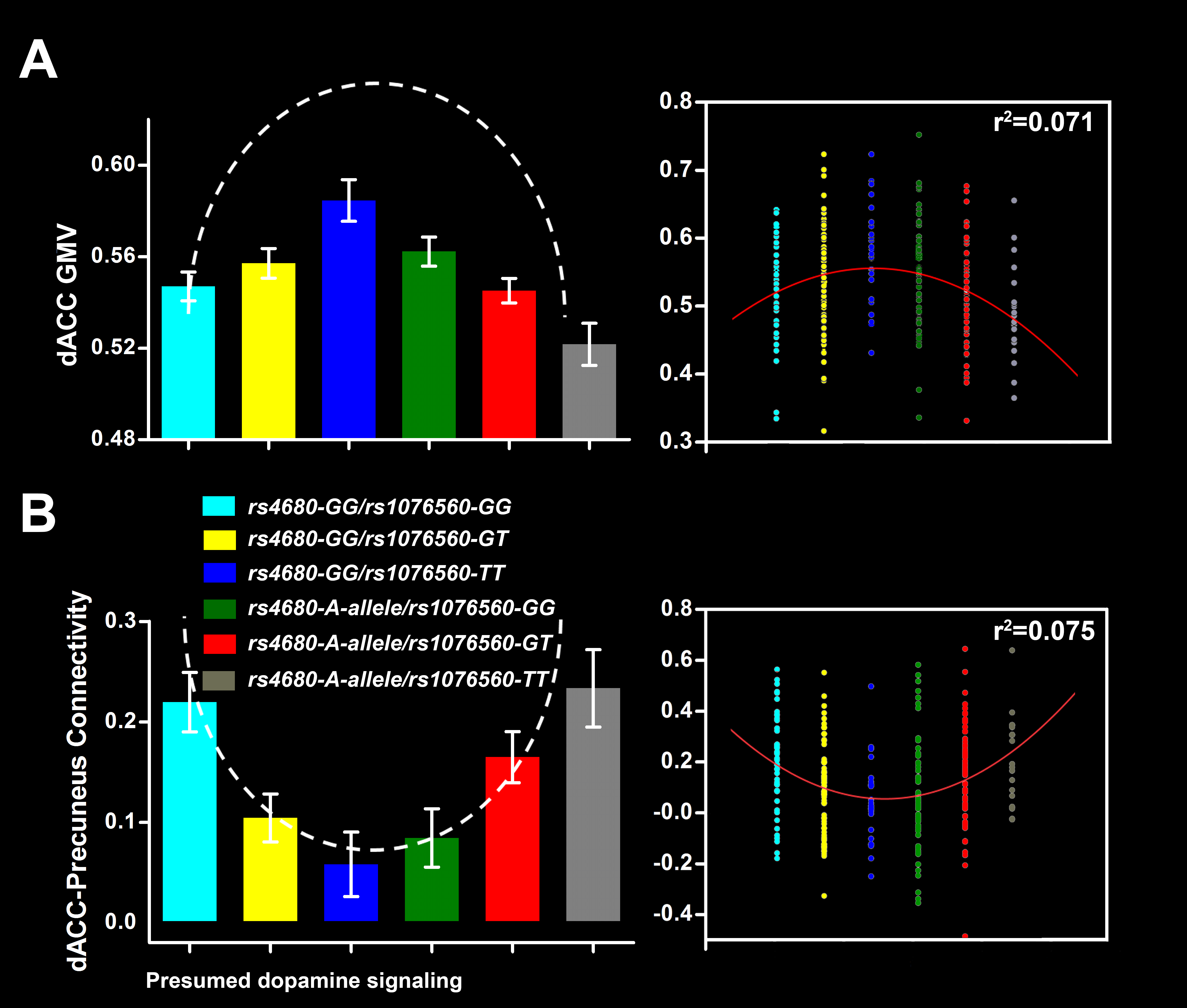


**Figure S2.** **The modulation effect of presumed dopamine signaling on GMV (A) and rsFC (B) of the dACC by sorting genotypes according to *COMT*.** The dashed line represents the presumed dopamine modulation pattern on GMV of the right dACC (inverted U-shape) and its rsFC with precuneus (U-shape). The red line was created by curve ﬁtting using quadratic regression. COMT, catechol-O-methyltransferase; GMV, gray matter volume; dACC, dorsal anterior cingulate cortex; rsFC, resting-state functional connectivity.


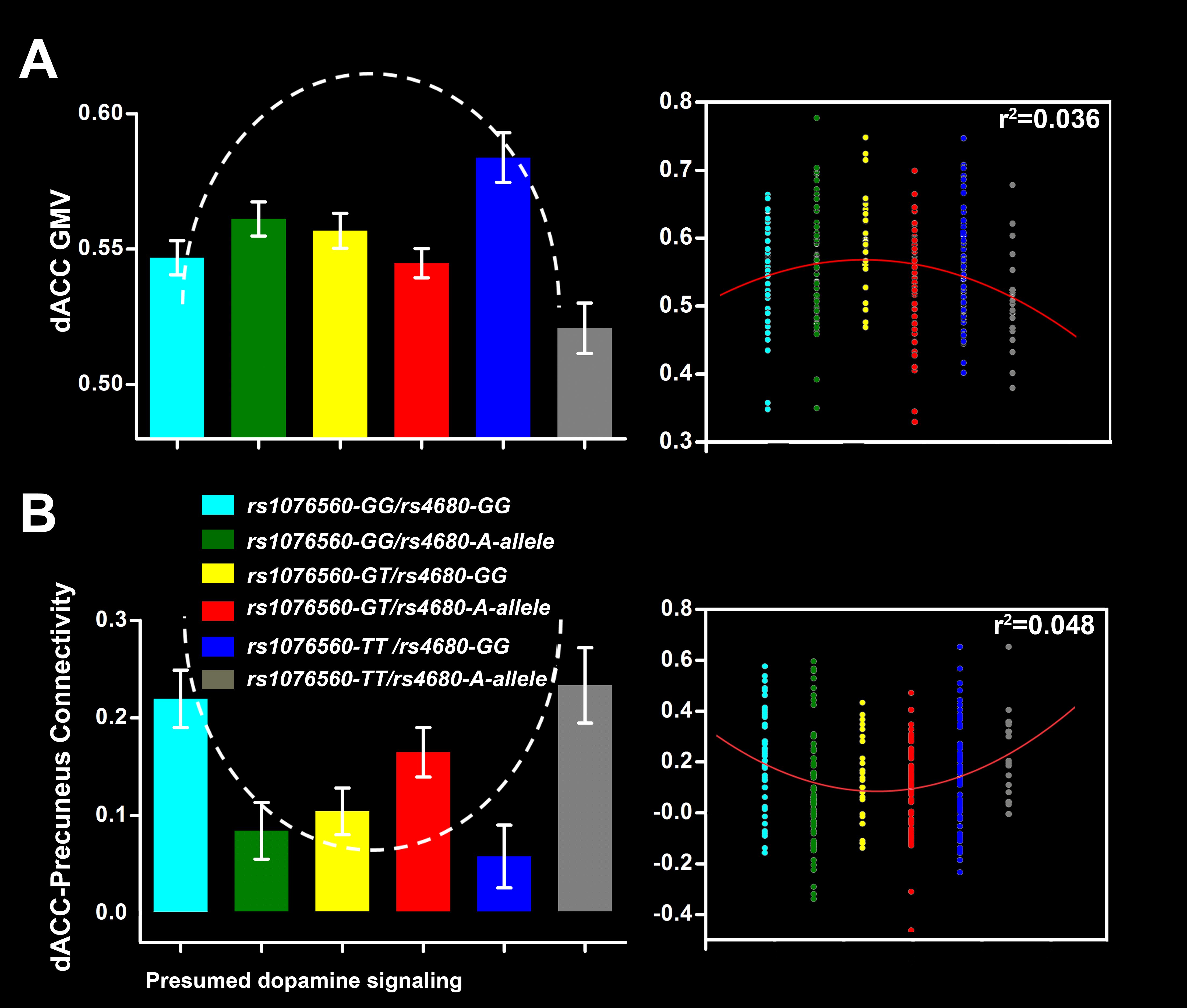


**Figure S3.** **The modulation effect of presumed dopamine signaling on GMV (A) and rsFC (B) of the dACC by sorting genotypes according to *DRD2*.** The dashed line represents the presumed dopamine modulation pattern on GMV of the right dACC (inverted U-shape) and its rsFC with the precuneus (U-shape). The red line was created by curve ﬁtting using quadratic regression. DRD2, D2 receptor gene; GMV, gray matter volume; dACC, dorsal anterior cingulate cortex; rsFC, resting-state functional connectivity.


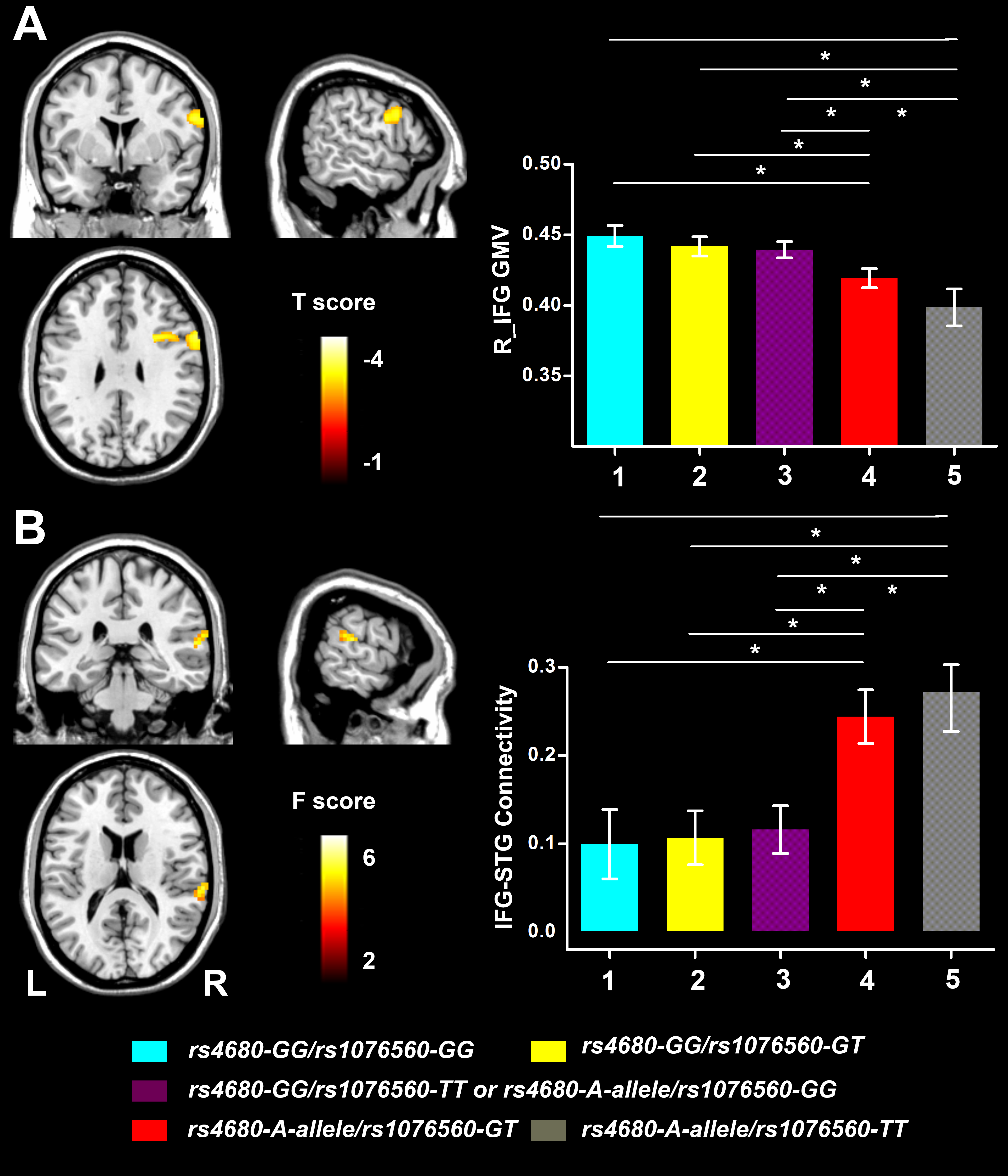


**Figure S4.** **Brain regions with additive effects of *COMT* and *DRD2* on GMV (A) and rsFC (B).** The horizontal axis of the bar plot represents five gene-gene cohorts based on the number of low-dopamine signaling associated alleles in *COMT (rs4680-G-allele)* and *DRD2 (rs1076560-G-allele).* COMT, catechol-O-methyltransferase; DRD2, D2 receptor gene; GMV, gray matter volume; IFG, inferior frontal gyrus; L, left; R, right; rsFC, resting-state functional connectivity; STG, superior temporal gyrus.


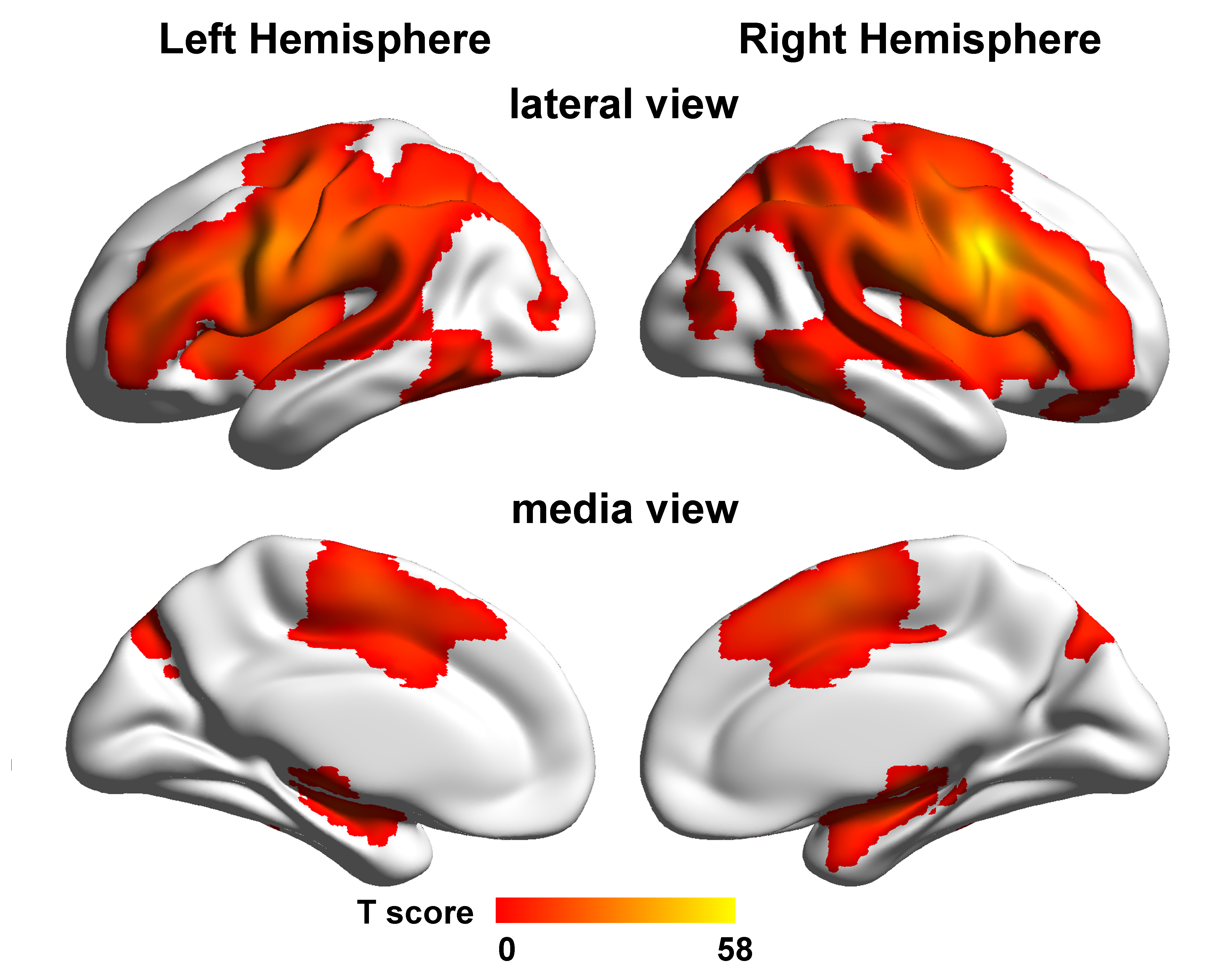


**Figure S5. The rsFC map of the right IFG**. One-sample t-test (FWE, *P*< 0.05) reveals that the right IFG is positively correlated with brain regions of the bilateral temporal and frontal lobes. FWE, family wise error; IFG, inferior frontal gyrus; rsFC, resting-state functional connectivity.


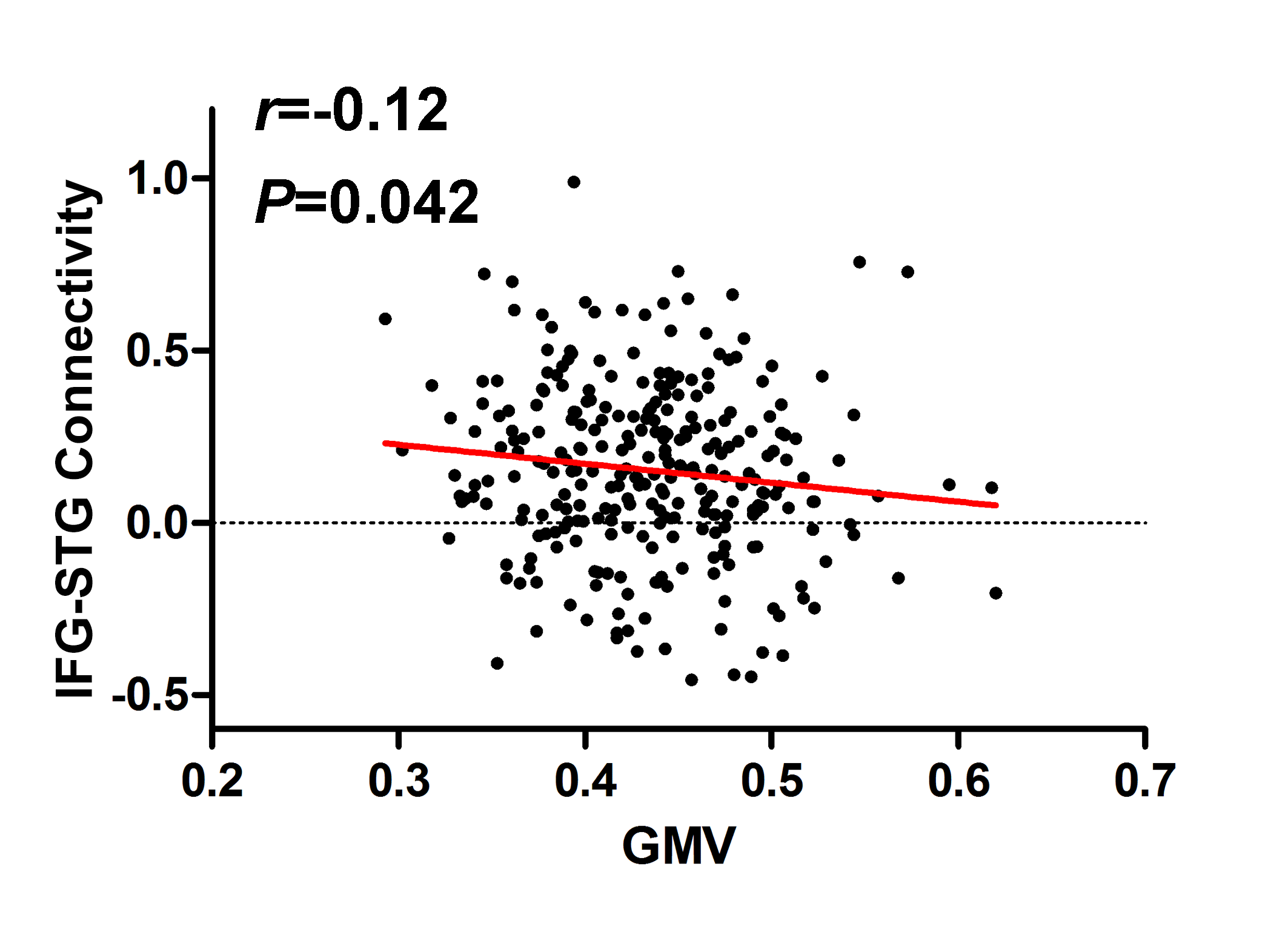


**Figure S6. Correlation between GMV of the right IFG and rsFC between the right IFG and STG.** GMV, gray matter volume; IFG, inferior frontal gyrus; rsFC, resting-state functional connectivity; STG, superior temporal gyrus.
