# Supplementary material for: Interactions of genetic variants reveal inverse modulation patterns of dopamine system on brain gray matter volume and resting-state functional connectivity in healthy young adults
Source: Brain Struct Funct. 2015 Oct 25;221(8):3891–901. doi: 10.1007/s00429-015-1134-4 (PMC5065899; doi:10.1007/s00429-015-1134-4)
Supplement: Supplementary file 2 — Supplementary material 2 (DOC 36 kb) [file 429_2015_1134_MOESM2_ESM.doc]

**Supplementary Materials**

**The additive interactions of *COMT* and *DRD2***

To test for additive effects, we created five gene–gene cohorts based on the number of low-dopamine signaling associated alleles in *COMT* (*rs4680-G-allele*) and *DRD2* (*rs1076560-G-allele*). Participants with *rs4680-GG/rs1076560-GG* (four “harmful” alleles) were classified into the “1 lowest-dopamine signaling” cohort; participants with *rs4680-GG/rs1076560-GT* (three “harmful” alleles) were classified into the “2 lower dopamine signaling” cohort; participants with *rs4680-GG/rs1076560-TT* or *rs4680-A-allele/rs1076560-GG* (two “harmful” alleles) were classified into the “3 moderate-dopamine signaling” cohort; participants with *rs4680-A-allele/rs1076560-GT* (only one “harmful” allele) were classified into the “4 higher-dopamine signaling” cohort.; and participants with *rs4680-A-allele/rs1076560-TT* (no “harmful” allele) were classified into the “5 highest-dopamine signaling” cohort. A voxel-wise linear regression was performed with the number of low-dopamine signaling associated alleles as the independent factor (1, 2, 3, 4 or 5) to assess GMV or rsFC differences.
