# Supplementary material for: Interactions of genetic variants reveal inverse modulation patterns of dopamine system on brain gray matter volume and resting-state functional connectivity in healthy young adults
Source: Brain Struct Funct. 2015 Oct 25;221(8):3891–901. doi: 10.1007/s00429-015-1134-4 (PMC5065899; doi:10.1007/s00429-015-1134-4)
Supplement: Supplementary file 3 — Supplementary material 3 (DOC 34 kb) [file 429_2015_1134_MOESM3_ESM.doc]

**Supplementary Results**

**The main effects of *COMT* on GMV**

The main effects of *COMT* genotypes on GMV (GRF correction at voxel level *P* < 0.005 and cluster level *P* < 0.005) are shown in Figure S1. Significant main effects of *COMT* on GMV were found in the right anterior cingulate cortex (ACC) (BA 32; peak MNI coordinate: x = 15, y = 25.5, z = 33; 1440 voxels; peak F = 14.23), the right precentral gyrus (BA 6; peak MNI coordinate: x = 64.5, y = 1.5, z = 27; 711 voxels; peak F = 16.00), the right inferior frontal operculum (IFop) (BA 44; peak MNI coordinate: x = 39, y = 12, z = 34.5; 459 voxels; peak F = 15.26), and the right mid-cingulate cortex (MCC) (BA 23; peak MNI coordinate: x = 18, y = -24, z = 46.5; 745 voxels; peak F = 13.12). Post-hoc testing showed that A-allele carriers (AA and GA) exhibited significantly smaller GMV in the four brain regions than the GG homozygotes (Figure S1). However, no significant main effect of *DRD2* on the GMV was found under the same statistical threshold (GRF correction at voxel level *P* < 0.005 and cluster level *P* < 0.005).
